# The first trimester human trophoblast cell line ACH-3P: A novel tool to study autocrine/paracrine regulatory loops of human trophoblast subpopulations – TNF-α stimulates MMP15 expression

**DOI:** 10.1186/1471-213X-7-137

**Published:** 2007-12-19

**Authors:** Ursula Hiden, Christian Wadsack, Nicole Prutsch, Martin Gauster, Ursula Weiss, Hans-Georg Frank, Ulrike Schmitz, Christa Fast-Hirsch, Markus Hengstschläger, Andy Pötgens, Angela Rüben, Martin Knöfler, Peter Haslinger, Berthold Huppertz, Martin Bilban, Peter Kaufmann, Gernot Desoye

**Affiliations:** 1 Department of Obstetrics and Gynecology, Medical University Graz, Austria; 2 Institute of Cell Biology, Histology and Embryology, Medical University Graz, Austria; 3 Institute of Anatomy, University Hospital RWTH Aachen, Germany; 4 Medical Genetics, Obstetrics and Gynecology, Medical University of Vienna, Austria; 5 Department of Obstetrics and Gynecology, Medical University of Vienna, Austria; 6 Department of Laboratory Medicine, Medical University Vienna & Ludwig Boltzmann Institute for Clinical and Experimental Oncology, Vienna, Austria

## Abstract

**Background:**

The trophoblast compartment of the placenta comprises various subpopulations with distinct functions. They interact among each other by secreted signals thus forming autocrine or paracrine regulatory loops. We established a first trimester trophoblast cell line (ACH-3P) by fusion of primary human first trimester trophoblasts (week 12 of gestation) with a human choriocarcinoma cell line (AC1-1).

**Results:**

Expression of trophoblast markers (cytokeratin-7, integrins, matrix metalloproteinases), invasion abilities and transcriptome of ACH-3P closely resembled primary trophoblasts. Morphology, cytogenetics and doubling time was similar to the parental AC1-1 cells. The different subpopulations of trophoblasts e.g., villous and extravillous trophoblasts also exist in ACH-3P cells and can be immuno-separated by HLA-G surface expression. HLA-G positive ACH-3P display pseudopodia and a stronger expression of extravillous trophoblast markers. Higher expression of insulin-like growth factor II receptor and human chorionic gonadotropin represents the basis for the known autocrine stimulation of extravillous trophoblasts.

**Conclusion:**

We conclude that ACH-3P represent a tool to investigate interaction of syngeneic trophoblast subpopulations. These cells are particularly suited for studies into autocrine and paracrine regulation of various aspects of trophoblast function. As an example a novel effect of TNF-α on matrix metalloproteinase 15 in HLA-G positive ACH-3P and explants was found.

## Background

In the first trimester of pregnancy the placental trophoblast has to fulfil a wide range of key functions in order to establish and maintain a successful pregnancy. These are not covered by one trophoblast phenotype, but rather associated with various trophoblast subpopulations each with unique features. In a series of differentiation steps the trophoblast subpopulations originate from cytotrophoblast stem cells [1], and acquire specific functions associated with their distinct tasks. Two main differentiation pathways of cytotrophoblasts are known: 1) in the villous pathway they differentiate and fuse to form the syncytiotrophoblast. This differentiation is paralleled by the onset of secretion of β-human chorionic gonadotropin (β-hCG). In the extravillous pathway they differentiate into the extravillous cytotrophoblast (EVT). A small subgroup of these cells maintains their proliferative capacity, while most of the cells further differentiate into highly invasive extravillous trophoblasts that invade the maternal uterine wall thereby ultimately opening the uterine spiral arteries.

Proper invasion is critical for placental and fetal development and its dysregulation results in a considerable spectrum of pregnancy abnormalities. Shallow invasion has been implicated in intra-uterine growth restriction (IUGR) [2,3] and early-onset pre-eclampsia [4-7]. In contrast, profuse invasion results in abnormally deep utero-placental adhesion such as seen in placenta accreta, increta and percreta, or may even lead to placental-site tumors [8]. The process of physiological invasion is tightly regulated in space and time by invasion-promoting and invasion-inhibiting factors that bind to receptors expressed on the extravillous trophoblasts. The invasion regulating factors either originate from the maternal decidua, the villous stroma or are secreted by various trophoblast populations. Decidua-derived soluble factors, such as transforming growth factor beta (TGFβ), tissue inhibitor of metalloproteinases-1 (TIMP-1) and kisspeptin-10 decrease trophoblast invasion, whereas insulin-like growth factor-I (IGF1) and-II (IGF2), β-hCG, endothelin-1 (EDN1), epidermal growth factor (EGF) and hepatocyte growth factor (HGF), all of them highly expressed in the placenta of the first trimester, stimulate trophoblast invasion [9-19]. TNF-α is a cytokine produced and secreted by trophoblasts and decidual macrophages that limits trophoblast invasion as well as the trophoblast-endothelial interaction in the uterine spiral arteries . Cytokines and growth factors secreted by cells in the decidua or the trophoblast thus form autocrine or paracrine loops ultimately fine-tuning the regulation of trophoblast invasion in a concerted manner.

*In vitro *research into the regulation of this physiological, non-malignant invasion is not only hampered by the limited availability of first trimester placental tissue, but also by the limited lifespan of primary first trimester trophoblasts in culture as they differentiate and shift into stages of apoptosis . In order to overcome this problem a number of human choriocarcinoma cell lines have been established such as BeWo, JAR and JEG3 that display a long lifespan and high proliferation activity. However, these cells reflect a carcinogenic cell type and differ from primary trophoblasts in many regards. Choriocarcinoma cells are malignant, immortal cells exhibiting non-physiological carcinogenesis  and they have developed mechanisms enabling them to escape from control of normal trophoblast invasion  i.e., from decidual cytokines  or low oxygen . Thus, choriocarcinoma cell lines have to be considered critically as cell model for trophoblast invasion.

Several trophoblast cell lines have been established and characterized. All of them have different features and each of them may resemble a distinct trophoblast subpopulation. They are important for investigations on the specific trophoblast subpopulations. However, they are of limited value for studies into the paracrine/autocrine interplay between subpopulations of trophoblasts a) as they do not comprise the subpopulations, b) because of their differing genetic background, and c) because of the varying strategies used for their immortalization. Since the interaction between subpopulations is a fine-tuned mechanism even subtle differences in genetic background may play an important role.

In order to overcome these limitations we have designed a strategy allowing to establish an immortal first trimester cell line (ACH-3P). Immortalization was achieved by fusing primary first trimester trophoblast cells with the choriocarcinoma cell line AC1-1 .

First trimester trophoblasts isolated from human placentas essentially comprise two trophoblast subpopulations, villous cytotrophoblasts (VCT) and invasive extravillous trophoblasts . Both populations differ among other features in their expression of HLA-G. We hypothesized the presence of these subpopulations also in the hybrid cells. Different from all other cell lines they will have a syngeneic background.

After their development and rigorous characterization the ACH-3P cells were used to identify autocrine and paracrine loops related to trophoblast invasion and differentiation. Thereby, a novel TNF-α effect on the expression of the membrane bound matrix metalloproteinase *MMP15 *(MT2-MMP) was discovered. MMP15 is mainly expressed by extravillous trophoblasts, cleaves components of the extracellular matrix and activates various pro-proteins present in the extracellular space i.e., proMMP2 and proTNF-α 

## Results

### Morphology and cell culture

**Figure 1:**
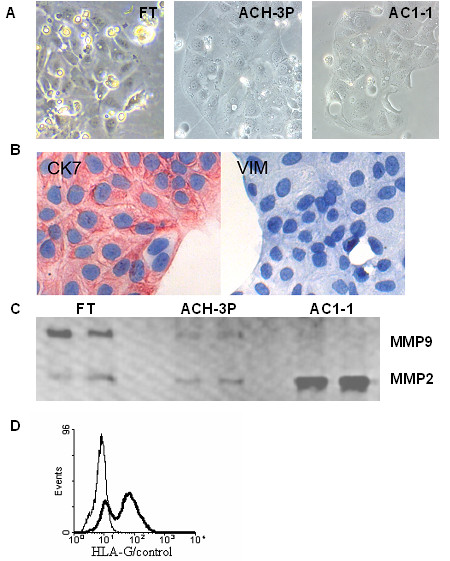
**Morphology and trophoblast marker expression of parental fusion partners and hybrid cells**. The photomicrographs representative shows cultured first trimester trophoblasts (FT) 48 hours after isolation, ACH-3P hybrid cells and AC1-1 choriocarcinoma cells, both in the 6th passage (20 × magnification) (A). IHC staining for cytokeratin-7 and vimentin of ACH-3P (B). Representative zymography of cell culture supernatants from FT, ACH-3P and AC1-1 after 48 h culture indicating presence of MMP9 and MMP2 (C). Representative FACS analysis for HLA-G (thick line) on viable ACH-3P cells (D). The result of the control antibody is shown (thin line) as overlay.

ACH-3P showed a uniform polygonal, epithelial-like cytomorphology (Fig. 1A) similar to the parental cell line AC1-1. The mean diameter and the volume of the cells were greater in both cell lines AC1-1 and ACH-3P than in primary trophoblasts (Table 1).

**Table 1:** Cellular size distribution and generation time of first trimester primary trophoblasts, ACH-3P and AC1-1 obtained by CASY^®^.

	**FT**	**ACH-3P**	**AC1-1**
**mean diameter [μm]**	**15.5 **± 1.2	**23.5 **± 1.0	**22.9 **± 0.7
**mean volume [μl]**	**2.6 **± 0.57	**8.8 **± 1.01	**8.1 **± 1.09
**generation time [h]**	**--**	**14.8 **± 1.48	**18.0 **± 1.85

The generation time in ACH-3P was shorter than in AC1-1, hence, ACH-3P, like AC1-1, represents a strongly proliferating cell line (Table 2). Most preparations of primary trophoblasts hardly proliferate under the conditions used and were, therefore, not included into this comparison.

**Table 2:** Primer pairs designed for semiquantitative RT-PCR. The number of PCR cycles is indicated for ACH-3P cells and first trimester trophoblasts.

**Gene**	**RefSeq**	**Primer**	**Cycle No**
**RPL30**	NM_000989	for: CCT AAG GCA GGA AGA TGG TG rev: CAG TCT GTT CTG GCA TGC TT	ACH-3P: 20 FT: 25
**HLAG**	NM_002127	for: AGG AGA CAC GGA ACA CCA AG rev: CCT CCA GGT AGG CTC TCC TT	ACH-3P: 25 FT: 27
**ITGA1**	NM_181501	for: GTC CAG TTG GGA GAG GTG AA rev: TGA ATG CCT CCT TTC TTG CT	ACH-3P: 26
**ITGA5**	NM_002205	for: TCC TGG AGT CCT CAC TGT CC rev: TCC TTG GCA GTA ACC CTG TC	ACH-3P: 23
**ITGA6**	NM_000210	for: GGC GGT GTT ATG TCC TGA GT rev: CAT GCT CAG TCT CTC CAC CA	ACH-3P: 25
**MMP2**	NM_004530	for: TTG GCA GTG CAA TAC CTG AA rev: GAG CAA AGG CAT CAT CCA CT	ACH-3P: 20
**MMP9**	NM_004994	for: GAG ACC GGT GAG CTG GAT AG rev: CAC CAA ACT GGA TGA CGA TG	ACH-3P: 35
**MMP14**	NM_004995	for: AAG GCA CAC TTG CTC CTG TT rev: CAC TGG TGA GAC AGG CTT GA	ACH-3P: 23 FT: 25
**MMP15**	NM_002428	for: GGC CGA CAT CAT GGT ACT CT rev: GTC AAC GTC CTT CCA CTG GT	ACH-3P: 24 FT: 26
**EDN1**	NM_001955	for: CTC GTC CCT GAT GGA TAA A rev: TCC TGC TTG GCA AAA ATT C	ACH-3P: 35 FT: 35
**EDNRA**	NM_001957	for: ATC ACC GTC CTC AAC CTC TG rev: CCA ATT CCC TGA ACA CGA CT	ACH-3P: 35 FT: 35
**EDNRB**	NM_000115	for: ATC TGC GAA TCT GCT TGC TT rev: AGC CAC CAA TCT TTT GCT GT	ACH-3P: 33 FT: 35
**β-hCG**	NM_000737	for: GTC AAC ACC ACC ATC TGT GC rev: GGC CTT TGA GGA AGA GGA GT	ACH-3P: 24
**IGF1**	NM000618	for: TGG ATG CTC TTC AGT TCG TG rev: CCT GCA CTC CCT CTA CTT GC	ACH-3P: 35 FT: 35
**IGF2**	NM_000612	for: GGT GCT TCT CAC CTT CTT GG rev: GGG GTA TCT GGG GAA GTT GT	ACH-3P: 25 FT: 25
**IGF1R**	NM_000875	for: GGA TGC GGT GTC CAA TAA CT rev: TGG CAG CAC TCA TTG TTC TC	ACH-3P: 29 FT: 32
**IGF2R**	NM_00876	for: TGA GTA TGC CTG CCA CAG AG rev: TCA AGG TGA GGT CTC CAT CC	ACH-3P: 26 FT: 29
**INSR**	NM_000208	for: GAG TCC TCG TTT AGG AAG ACG rev: AAG TGT TGG GGA AAG CTG CCA	ACH-3P: 31 FT: 33
**TNFA**	NM_000594	for: GAC AAG CCT GTA GCC CAT GT rev: TTG ATG GCA GAG AGG AGG TT	ACH-3P: 33 FT: 33
**TNFR1**	NM_001065	for: ACC AAG TGC CAC AAA GGA AC rev: CTG CAA TTG AAG CAC TGG AA	ACH-3P: 25 FT: 27
**TNFR2**	NM_001165	for: CCA AGT GGT TTC CAA GGT GT rev: CAC GGC AGC ATT AAT CAC AG	ACH-3P: 29 FT: 31

### Expression of trophoblast markers

Immunocytochemistry and FACS analysis of ACH-3P (Table 1) demonstrated a stable expression of the trophoblast marker cytokeratin-7, but absence of vimentin, a protein, that is present in all non-trophoblast cells. This is a clear demonstration of the trophoblast origin of ACH-3P (Fig. 1B).

ACH-3P secreted hCG, a hormone expressed by differentiated cytotrophoblasts and the syncytiotrophoblast, but accumulated over 24 hours higher in AC1-1 and highest in first trimester trophoblasts (Table 3). According to gelatin gel zymography ACH-3P produce the invasion relevant active matrix-metalloproteinases MMP2 and MMP9 similar to primary trophoblasts, whereas in the culture supernatant of AC1-1 a very high amount of only MMP2 was detected (Fig. 1C).

**Table 3:** Expression of trophoblast markers and markers for trophoblast subpopulations in primary first trimester trophoblasts, ACH-3P and AC1-1. The expression of the trophoblast marker cytokeratin-7 (CK7) was determined by immunocytochemistry and of markers for trophoblast subtypes (HLA-G, integrins) were detected by FACS analysis. Secretion of hCG was measured after 24 hours in the culture supernatant and normalized to the cell number. The activity of MMP2 and MMP9 was measured by gelatin gel zymography. + indicates ~20–40% of cells. ++ indicates ~40–90%; +++ indicates 100% of cells; – indicates no expression.

	**FT**	**ACH-3P**	**AC1-1**
CK7	+++	+++	+++
Vimentin	-	-	-
HLA-G	++/-	++/-	++
integrin α6	+	++	+
integrin β4	+	++	++
integrin α5β1	+/-	++	++
integrin α1	+/-	-	-
hCG	+++	+	+
MMP2	++	+	+
MMP9	+	+	+++

According to FACS analysis ACH-3P cells are heterogeneous for the non-classical MHC antigen HLA-G, a protein characteristic for highly invasive EVT. About 60% of the cells were HLA-G negative and 40% showed moderate HLA-G expression (Fig. 1D). This heterogeneity was also found in first trimester primary trophoblasts, but with a smaller number (12%) of HLA-G positive cells. In contrast AC1-1 cells strongly express HLA-G. According to propidium iodide (PI) staining after methanol fixation the HLA-G positive and negative population within the ACH-3P cells did not differ in DNA amount (data not shown).

### Invasion ability

**Figure 2:**
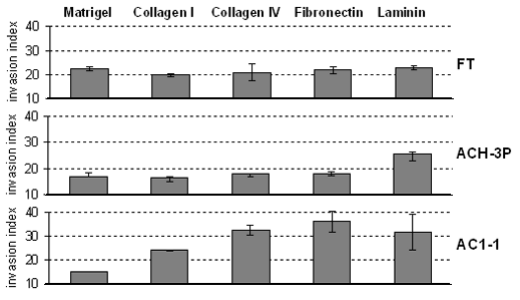
**Comparison of invasion indices of parental fusion partners and hybrid cells**. Invasion indices (mean ± SD, n = 3) of first trimester trophoblasts (FT), ACH-3P and AC1-1 after 48 h in the presence of 10% FCS on different extracellular matrices.

In invasion assays primary trophoblasts and ACH-3P revealed the highest invasion index on laminin and the lowest on collagen-1 (Fig. 2). A different pattern was observed with the highly invasive AC1-1. Here, invasion was strongest on fibronectin and weakest on Matrigel.

### Cytogenetic analysis

**Figure 3:**
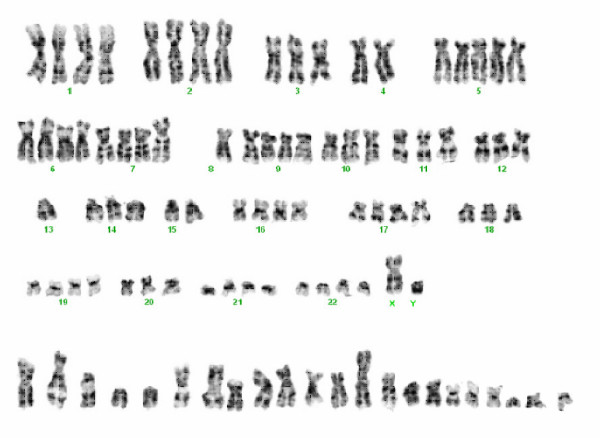
**Representative karyotype of ACH-3P cells**. Chromosomes given in the bottom row could not be assigned.

Fifty metaphases were cytogenetically analysed (Fig. 3). Fusion of AC1-1 cells that contain 60–69 chromosomes with primary trophoblasts resulted in ACH-3P cells. They contain 94–98 chromosomes and represent a male cell line like their parental cell line AC1-1.

### Microarray analysis

**Figure 4:**
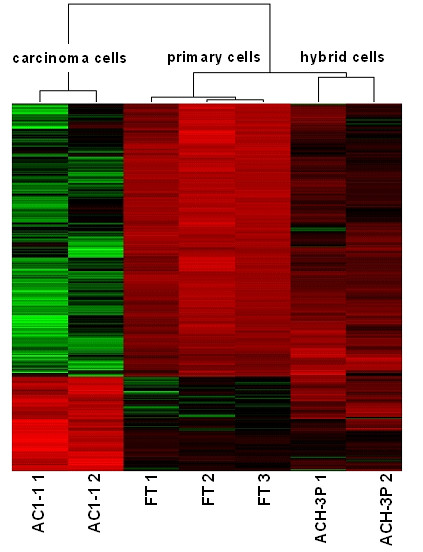
**Heatmap analysis of microarray data showing hierarchical clustering of 832 differentially expressed genes between first trimester primary trophoblasts, AC1-1 and ACH-3P cells**. Three first trimester primary trophoblast preparations (FT 1, 2, 3), two AC1-1 (AC1-1 1, 2) and two ACH-3P (ACH-3P 1, 2) RNA preparations were analyzed. Red or green colours indicate differentially up- or downregulated (619) genes, respectively (FC > 2 fold). The dendrogram displayed on top was based on hierarchical clustering of the samples using the average linkage method indicating the strong relation of the ACH3P cell line with the primary cells.

A heatmap displays all genes that were differentially expressed (832 genes with a fold change > 2) between these cells (Fig. 4). The dendrogram at the top indicates the close relationship between ACH-3P and first trimester primary trophoblast cells and the larger distance to the AC1-1 cells. Thus, the transcriptome of the fused ACH-3P closely resembles that of the parental primary first trimester trophoblasts.

When the genes differentially expressed between the ACH-3P and primary trophoblasts were clustered according to their putative function, a predominant proportion was related to cell cycle regulation, oncogenesis and proliferation. The full list as well as a functional categorization into most prominent biological processes of these genes is given in the additional tables [1 and 2]. The carcinoma cells AC1-1 differ by more than 800 genes from the ACH3P and the primary trophoblasts [see figure 1].

### Characteristics of the HLA-G positive and negative ACH-3P and primary trophoblast subpopulations

**Figure 5:**
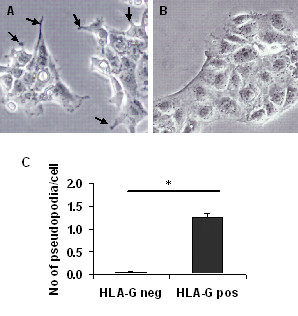
**Photomicrograph of HLA-G positive (A) and HLA-G negative (B) subpopulation of ACH-3P cells**. Cells are shown in the 3rd passage after immuno-separation (20 × magnification). Please note the presence of pseudopodia (indicated with arrows) in the HLA-G expressing (A) but not in the HLA-G negative cells. The number of pseudopodia per cell is shown in C. 200 cells of the outermost cells within the clusters were counted. *p > 0.001

Immuno-separation of the HLA-G positive and negative ACH-3P cells resulted in morphologically different cell populations (Fig. 5). More (p > 0.001) HLA-G positive than negative cells (1.3 vs. 0.05) showed a higher number of pseudopodia. RT-PCR found EVT-specific genes expressed at higher levels in the HLA-G positive subpopulation. Examples include *HLA-G*, *ITGA5*, *MMP2*, *MMP9*, but also other MMPs (*MMP15*), a pro-invasive acting receptor (*IGF2R*) of which *β-hCG *modulates the affinity. The expression of ITGA1 and ITGA6 between the HLA-G positive and negative ACH-3P subpopulations did not correlate with the expression pattern of HLA-G positive and negative primary cells. This degeneration is obviously a remnant of the parental AC1-1 cells and a common result of carcinogenesis (Table 4). No change was found in the expression of the endothelin and IGF1 receptor-ligand systems (EDN1; EDNRA; EDNRB; IGF1; IGF2; IGF1R). The difference in gene expression levels between HLA-G expressing and lacking ACH-3P resembled the HLA-G positive and negative subpopulations of the primary trophoblasts. MMP15 and IGF2R were higher expressed also in their HLA-G positive ACH-P and trophoblast subpopulation whereas the TNFR2 mRNA level was considerably higher in the HLA-G negative subpopulations of cell line (3.5 ± 0.4) and primary cells (1.3 ± 0.18). The difference in expression of the investigated genes remained stable in the two ACH-3P subpopulations up to passage 5 after separation after which time these decline.

**Table 4:** Expression of markers for trophoblast subpopulations and invasion relevant ligand-receptor systems in HLA-G positive versus HLA-G negative ACH-3P, and in HLA-G positive versus HLA-G negative primary trophoblasts (i.e. EVT vs. VCT). Expression was measured by RT-PCR. The fold change values refer to the HLA-G negative cells (= 1). Direction of expression changes of well studied genes in EVT versus VCT are indicated with arrows and according to literature. Because of the low yield of primary trophoblast preparations only genes with differential expression between the ACH-3P subpopulations were investigated. Genes that are differently expressed between the HLA-G positive and HLA-G negative cells in primary trophoblasts and in the ACH-3P cells are printed in bold. (mean ± SD of n = 4 experiments). NC: no change

	**ACH-3P**	**FT**
	fold change **HLA-G + vs. HLA-G-**	fold change **EVT vs. VCT**

HLA-G	20 ± 0.50	7.7 ± 0.30
ITGA1	NC	↑ [1]
**ITGA5**	1.2 ± 0.10	↑ [1]
ITGA6	NC	↓ [1]
**MMP2**	3.9 ± 0.50	↑ [1]
**MMP9**	5.3 ± 0.70	↑ [1]
**MMP15**	3.0 ± 0.40	2.3 ± 0.10
**β-hCG**	4.4 ± 0.20	↑ [50]
IGF2	NC	NC
**IGF2R**	1.2 ± 0.10	1.2 ± 0.05
TNFA	NC	NC
TNFR1	NC	NC
**TNFR2**	-1.3 ± 0.18	-3.5 ± 0.40

### Effect of TNF-α on *MMP15 *expression in HLA-G positive and negative ACH-3P cells and in first trimester placental explants

**Figure 6:**
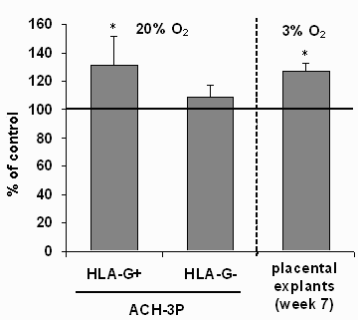
**TNF-α treatment (10 ng/ml) increased MMP15 mRNA expression in the HLA-G positive ACH-3P cells and in first trimester placenta explants**. Please note that the oxygen conditions for cell culture and explant culture are different. Data (mean ± SD of n = 4 experiments) are expressed relative to the untreated controls (= 100%). Data shown were normalized to the controls whereas the statistic tests used the raw data. *p < 0.05 vs. control

TNF-α treatment increased the expression of *MMP15 *only in the HLA-G positive ACH-3P cells and in first trimester placental explants (Fig. 6). The oxygen tension (3% O_2_) used for the explant culture mimics the *in vitro *situation in a 7th week of gestation. As EVT are the cell type predominantly expressing MT2-MMP , the MT2-MMP in the explant is presumed to be expressed by the EVT.

## Discussion

### ACH-3P closely resemble first trimester trophoblasts

The new hybrid cell line ACH-3P strongly expresses cytokeratin-7, but is devoid of vimentin, a protein expressed in all non-trophoblast cells of the placenta proper . Hence, ACH-3P is a trophoblast cell line. In addition to this standard criterion, the expression pattern of well established proteins [1] typical for different first trimester trophoblast subpopulations (Table 4) demonstrates that ACH-3P cells express various markers for invading EVT i.e., HLA-G , integrin α5β1  and the gelatinases MMP2 and MMP9 . In addition, the presence of markers typical for the proliferating cytotrophoblasts (integrin α6β4) and the villous syncytiotrophoblast (hCG) in ACH-3P cells obviously reflects the heterogeneous pool of trophoblast subpopulations in the preparations of primary first trimester trophoblast cells (Table 4). As the ACH-3P comprise a polyclonal cell line, the presence of other subpopulations in addition to the described HLA-G positive and negative cannot be excluded, but these may only occur to a low proportion. After their separation, the subpopulations remain stable up to the 5th passage. Therefore, it is mandatory to always freshly isolate the target subpopulation from the polyclonal pool for each experiment.

In addition to the presence of the characteristic EVT markers, ACH-3P were also capable of invading extracellular matrices. The invasion indices on the different matrices resembled that of first trimester trophoblasts with highest values on laminin, which is paralleled by a strong expression of the laminin receptor integrin α6. Hence, ACH-3P contain a subpopulation of cells with an EVT phenotype. Contrary to the invasion properties and the expression of trophoblast markers, the morphology, cellular size and proliferation of ACH-3P resembled that of the parental choriocarcinoma cell line AC1-1, a favourable result as proliferation is a desired property of a model cell line.

Cytogenetic analysis revealed a high number of chromosomes. This was expected due to the fusion of a normal somatic cell type with a tumour cell line that per se contains 60–69 chromosomes. The range of 94 to 98 chromosomes and the stable DNA content of ACH-3P observed in FACS analyses proved homogeneity with regard to chromosome number and thus the consistent fusion of the hybrid cells.

Microarray analysis paralleled the above observed close relationship between first trimester primary trophoblasts and ACH-3P. This was clearly seen by the hierarchical clustering of all genes differently expressed between ACH-3P and first trimester primary trophoblasts as well as between first trimester primary trophoblasts and AC1-1.

### Autocrine/paracrine regulation of invasion relevant molecules of ACH-3P subpopulations

**Figure 7:**
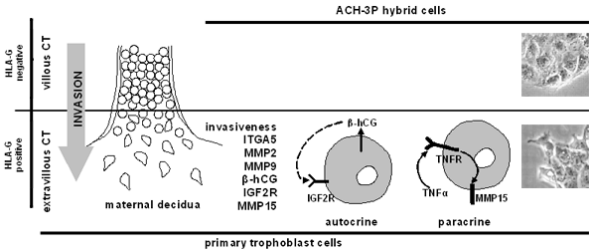
**Comparison between HLA-G negative and positive trophoblasts as well as HLA-G negative and positive ACH-3P subpopulations**. Both HLA-G positive subpopulations express a higher amount of *ITGA5*, *MMP2*, *MMP9*, β-hCG, *IGF2R and MMP15 *than their syngeneic, HLA-G negative counterpart. The higher β-hCG secretion and *IGF2R *expression suggest the presence of an autocrine loop stimulating invasion. Furthermore, the strong effect of TNFα on the up-regulation of *MMP15 *expression in HLA-G positive cells suggests modulation of trophoblast invasion and/or action by TNFα.

After immuno-separation for HLA-G the two subpopulations differed in their morphology and in the expression of a number of invasion relevant genes. The HLA-G positive cells impress by their high number of pseudopodia, extensions of the plasma membrane that enable invasion of motile cells. This obviously reflects the high invasion capacity of the HLA-G positive ACH-3P subpopulations. Additionally, these cells exhibited a higher expression of various markers of the invasive EVT lineage such as integrin α5, MMPs (i.e. MMP2, MMP9, and MMP15) and hCG. This strongly resembled the HLA-G positive first trimester trophoblasts indicating that the phenotype of the trophoblast subpopulations remained in the fused cells (Fig. 7).

This notion is substantiated further by the prevailing presence of components forming an established autocrine loop to regulate invasion in the HLA-G positive primary trophoblasts and ACH-3P cells. Beta-hCG is known to stimulate invasion by modulating IGF2 binding to the IGF-2R on [12]. The higher expression of both modulator and receptor in the HLA-G positive EVT was confirmed in our primary trophoblast preparations. It was also found in the ACH-3P cells (Table 4; lower panel). This demonstrates that the hybrid cells are well-suited for studies into the regulation of components making up autocrine or paracrine loops.

These features of ACH-3P cells were then used to test the hypothesis that TNF-α, presumably derived from the decidual macrophages or trophoblasts, will stimulate MMP15 expression on invading i.e., HLA-G positive EVT. If confirmed this would identify a novel effect of TNF-α and establish a new heterologous paracrine or autocrine regulation of one factor involved in trophoblast action (Fig. 7). MMP15 is stronger expressed in the HLA-G positive primary cells (350%) and ACH-3P (30%), respectively, and TNF-α is produced by cells located nearby the invading trophoblasts i.e., the decidual macrophages. This suggests a direct interaction and regulatory loop between TNF-α and MMP15 expression. Furthermore, MMP15 was up-regulated by TNF-α only in the invasive HLA-G positive subpopulations of ACH-3P although the expression of *TNFR2 *was higher in the HLA-G negative subpopulations of the cell line and the primary cells. The stronger responsiveness of *MMP15 *expression towards TNF-α in the HLA-G positive cells despite their lower *TNFR2 *expression is most likely a result of their higher basal *MMP15 *expression level. The up-regulation was also found in first trimester placental explants, an experimental model close to the *in vivo *situation. These findings highlight that the induction of MMP15 expression is a physiological effect and not only a result of the specific conditions of cell culture. Because the different experimental models i.e., ACH-3P cell culture and placental explants, showed a similar response (Fig. 6), the effect of TNF-α on MMP15 expression is obviously independent of oxygen concentration and extracellular matrix. Importantly, again the model cells resembled the original tissue. The consequences of these results for invasion regulation are unclear, because *in vitro *TNF-α limits trophoblast invasion. Nevertheless, MMP15 may not only be involved in processes related to invasion, but as a result of its wide spectrum of substrates, also in activation of pro-proteins such as proMMP2 and proTNF-α. The newly identified induction of MMP15 expression by TNF-α thus further supports a key role of TNF-α in regulation of trophoblasts.

## Conclusion

Collectively, the new ACH-3P cell line provides the characteristics of different aspects of first trimester trophoblast subtypes combined with the proliferation potential of the parental choriocarcinoma cell line. It therefore, represents a useful tool for the investigation of characteristic features of first trimester trophoblasts such as non malignant invasion. Furthermore, two subpopulations can be separated according to HLA-G expression. The HLA-G positive cells strongly resemble the invasive EVT and thus represent a tool to investigate trophoblast invasion against the syngeneic HLA-G negative background. These cells are particularly well-suited for studies into autocrine and paracrine regulation of various aspects of trophoblast function. As an example a novel effect of TNF-α on MMP15 expression in HLA-G positive first trimester trophoblasts and ACH-3P was found (Figure 7).

## Methods

### Cell lines

The mutant choriocarcinoma cell line AC1-1 was derived from JEG3 by mutagenesis and subsequent cloning of mutants defective for hypoxanthin-guanin-phosphoribosyl-transferase .

### Isolation of primary trophoblast cells from first trimester placenta

The study was approved by the Ethical Committee of the Medical University of Graz.

Primary trophoblasts were isolated from first trimester placentas (weeks 11 and 12, n = 12) after pregnancy terminations for psychosocial reasons as described previously .

Briefly, chorionic villi were digested for 15 min at 37°C in a digestion mix containing trypsin (0.125%, Gibco, Paisley, UK) dispase/Ribonuclease A (2.4 U/ml dispase, Roche Diagnostics, Mannheim, Germany; 0.013% Ribonuclease A, Sigma Chemicals, St. Louis, USA). The dispersed cells were separated from the residual tissue by filtration through a cell sieve, and applied on top of a Percoll (Pharmacia, Uppsala, Sweden) density gradient ranging from 10% (v/v) to 70%. After centrifugation, the band containing the trophoblasts was collected. Cells were further purified by incubation with immuno-magnetic beads (Dynabeads M-450, Dynal) conjugated with CD45 RB Leukocyte Common antigen (Dako, CA) and, subsequently, incubated with beads conjugated with the fibroblast-specific antibody A 502 (Dianova, Hamburg, Germany). This procedure removes all non-trophoblastic components . Primary trophoblasts were cultured in DMEM (Gibco) supplemented with 10% FCS in a humified atmosphere of 5% CO_2 _at 37°C for 48 h. The cells thus obtained represent a mixture of both trophoblast subpopulations i.e., villous and extravillous trophoblasts.

### Cell fusion

Immortal AC1-1 cells and primary trophoblast cells were fused as described. Cells were detached by a 0.25% trypsin solution, washed and equal numbers of AC1-1 cells and trophoblasts were mixed and pelleted. One ml of a prewarmed solution containing 30% v/v polyethylene glycol (Roth, Darmstadt, Germany) and 10% v/v dimethylsulfoxide in Ham's F-12 (Gibco) was added drop wise over a period of 1 min. This procedure was repeated twice. Subsequently, 7 ml of Ham's F-12 was added, the resulting cell suspension was pelleted and resuspended in Ham's F-12 with 10% FCS. Every two passages, the cells were cultured in selection medium containing azaserin (5.7 μM) and hypoxanthine (100 μM).

### Proliferation and size distribution

For the determination of the generation time i.e., the time between two dividing steps of a single cell, AC1-1 and ACH-3P cells were grown in 24 well plates with Ham's F-12 medium supplemented with 10% FCS (HiClone). After 12, 36 and 60 hours trypsinized cells were counted using a CASY^® ^Cell Counter (Schärfe System, Germany) that concomitantly measures the cellular size distribution. The experiment was run in triplicate.

### Secretion of hCG

First trimester trophoblasts, ACH-3P and AC1-1 were grown in their appropriate culture medium (DMEM and Ham's F-12) supplemented with 10% FCS. The concentration of hCG was determined in the culture medium (Dade Behring Inc., Deerfield, Illinois) after 24 and 48 hours  and normalized to the cell number at each time point.

### Immunocytochemistry

Cells were grown on chamber slides until confluency and then fixed with ice-cold acetone (Merck, Darmstadt, Germany) for 5 min. The presence of cytokeratin-7 (Clone OV-TL 12/30; 1:200; Dako) and vimentin (Clone V9; 1:500; Dako) was detected by the antiperoxidase method (Lab vision, Fremont, CA). Slides were washed in TBS (tris buffered saline) pH 7.5 with 0.05% Tween 20 (Sigma, St. Louis, MO) for 5 min. Non-specific binding sites were blocked with UV block (Lab vision) for 10 min. Subsequently, the primary antibody (diluted in Dako antibody diluent) was applied for 30 min. After three washings in TBS/Tween the secondary antibody (goat anti polyvalent, Lab vision) was applied for 10 min. Following further washings, the slides were incubated with streptavidin peroxidase (Lab vision) for 10 min and washed again. The chromogenic reaction was started by addition of AEC substrate system (Lab vision) for 4 min and washing in aqua dest. Haemalaun (Sigma) solution was used for nuclear counterstaining. Slides incubated with mouse unspecific immunoglobulin fractions (Mouse IgG1 negative control, Dako) in the same concentration as the primary antibody served as negative controls.

### Analysis by flow cytometry

#### a) Staining of cells

ACH-3P and AC1-1 cells were trypsinized from subconfluent cultures. Primary trophoblast cells were trypsinized from adherent cultures 24 hours after isolation. Cells were centrifuged for 5 min at 3000 rpm and 20°C, washed in washing buffer phosphate-buffered saline (PBS) with 0.25% bovine serum albumin and 2 mM EDTA, divided into portions of 1–4 × 10^5 ^cells each, and centrifuged again. For staining of membrane antigens on living cells the following antibodies were used: anti-integrin α1 (Clone FB12; 1:500; Chemicon), anti-integrin α6 (Clone GoH3; 1:500; BD Pharmingen), anti-integrin β4 (BD Pharmingen; 1:500), anti-integrin α5β1 (Dako; 1:25), and HLA-G (Clone MEM-G/9; 1:500; Abcam). For staining of cytoplasmatic antigens (cytokeratin-7; 1:500 and vimentin; 1:360) and DNA (with propidium iodide, PI) cell pellets were first resuspended in 0.5 ml 70% methanol in distilled water, -20°C, by repeatedly pipetting up and down. Cells were methanol-fixed for 10 min at 4°C, washed and centrifuged again. Cell pellets of live or methanol-fixed cells were resuspended in 100 αl blocking buffer (washing buffer with 5% FCS and 5% pooled human serum) containing the diluted primary antibody and incubated with agitation for 30 min at RT. Cells were washed again in 0.5 ml washing buffer, followed by incubation in 100 μl washing buffer containing the appropriate secondary antibody (rabbit anti-mouse-FITC, 1:200; goat anti-rat-Cy2, 1:25) for 30 min at RT with agitation. After further washing and centrifugation, cells were resuspended in 0.4 ml washing buffer. Buffered formalin, pH 7.0, was added up to 1% to stained live cells. Methanol-fixed cells were resuspended in washing buffer containing 10 μg/ml PI and stored without formalin.

#### b) FACS analysis

A FACScalibur (Beckton Dickinson, San Jose, CA, USA), with an Argon laser (488 nm) was used. Amplifiers for forward scatter (FSC) and sidescatter (SSC) were set such that the majority of events fitted inside a linear FSC/SSC dotplot. FITC staining was measured in channel 1 (FL1, bandpass 515–545 nm) with logarithmic amplification, DNA staining by PI was measured in channel 3 (FL3, bandpass >600 nm) with linear amplification. A total of 5000 events were acquired of each sample. Data were quantitatively analysed using CellQuest Pro software (Beckton Dickinson), and with WinMDI software, version 2.8 (Joseph Trotter, Scripps Research Institute, La Jolla, CA, USA) for producing plots for reproduction. The measured events were gated in an FSC/SSC plot to exclude subcellular fragments which have very low FSC and SSC and cell aggregates characterized by very high SSC. Also, cellular fragments lacking a nucleus (PI-negative) were gated out from methanol-fixed samples. Of each sample, the mean FITC- fluorescence intensity was plotted. The relative antigen expression level in a cell population was calculated by dividing the mean fluorescence measured after staining with a particular antibody by the mean fluorescence level measured after staining with the appropriate control antibody (Table 5). Antigen expression levels were therefore depicted as "fluorescence intensity relative to control". When a cell sample displayed a heterogeneous antigen expression profile, a positive and a negative population was isolated in separate gates. The proportion of cells expressing or lacking this antigen was calculated. Moreover the relative antigen expression levels for the positive and negative population was calculated separately in fluorescence intensities relative to control.

**Table 5:** Antibodies and their appropriate IgG controls used for FACS analysis.

**Host**	**Antigen**	**IgG subclass**	**Isotype control**
mouse	integrin α5β1	IgG3, κ	mouse IgG3
mouse	integrin β4	IgG1, λ	mouse IgG1
rat	integrin α6	IgG2a	rat IgG2a
mouse	integrin α1	IgG1	mouse IgG1
mouse	HLA-G	IgG1	mouse IgG1

### Zymography

Gelatin zymography was used to measure the activity of MMP2 and MMP9. One × 10^5 ^ACH-3P, AC1-1 and primary trophoblast cells were seeded in 12-well plates and grown in their appropriate culture medium (Ham's F-12 for the cell lines and DMEM for the primary cells) without FCS. After 24 and 48 h, the culture supernatant was collected and protein concentration was determined using the Bradford assay (Bio-Rad Laboratories). Two-fold concentrated sample buffer, 126 mmol/L Tris-HCl, 20% glycerol, 4% SDS, 0.005% bromphenol blue, was added 1:1 to the culture supernatants containing equal amounts of protein before loading on the 10% polyacrylamide gels supplemented with 1% gelatine (Invitrogen, Carlsbad, CA). Electrophoresis, renaturing and development of the gels were performed according to the manufacturer's instructions (Invitrogen). The developed gels were stained with 5% Coomassie blue solution and destained appropriately. The gels were photographed and the proteolytic activity was determined by densitometric analysis using the Alpha DigiDoc 1000 (Alpha Innotech, CA) software.

### Invasion Assay

Cell invasion activity was measured by a standard invasion assay [15] and expressed as invasion index. Matrigel, mouse laminin, collagen I and IV, and fibronectin were coated on inserts with 3 μm pores. Invasion chambers and inserts were purchased from Becton Dickinson, Bedford, MA. One × 10^6 ^cells of each cell type were plated with 5 ml DMEM onto 25 cm^2 ^tissue culture flasks and incubated with 50 μCi [^3^H]-5-methyl-thymidine overnight to prelabel the cells. After this incubation period, the adherent cells were washed twice with HBBS (Gibco) and removed with trypsin-EDTA. Cells were centrifuged at 300 × g for 7 min and resuspended in DMEM with 10% FCS. Five × 10^4 ^cells/500 μl media were plated onto the upper side of the membrane of each insert of the 24 well Invasion Chamber Assays. One ml DMEM was placed into the lower chamber. Cells were cultured in 4–8 parallel wells for 24 and 48 hours under 5% CO_2_/95% air atmosphere at 37°C. After each incubation period, the media in the upper and lower wells were removed and placed in separate vials. Trypsin-EDTA 1× was added to the upper wells to remove cells on the surface of the membrane. The removed cells were pooled with the media of the upper well. The membrane was carefully removed from the plastic and pooled with the media of the lower well. Radioactive disintegrations in the vials were counted in a scintillation counter (Beckman). The invasion index was calculated according to the formula: invasion index = radioactivity in lower well/radioactivity in (lower well + membranes + upper well) × 100.

### Cytogenetic analysis

The cultured cells were cytogenetically analysed according to the standard protocols. Chromosome banding was produced by means of the trypsin-Giemsa method.

### Microarray analysis

RNA was isolated from three different first trimester trophoblast preparations and two different passages of AC1-1 and ACH-3P, respectively, using Trizole (MRC, Ohio) followed by quality assessment using a Bioanalyzer (Agilent, CA). Experimental procedures and data analysis settings followed proposed standards. From 5 μg of RNA, cDNA was synthesized (SuperScript double stranded cDNA Synthesis Kit, Invitrogen, CA), *in vitro *transcribed (RNA Transcript Labeling Kit, Enzo diagnostics, Farmingdale, NY) and fragmented. To test the quality of the cRNA it was hybridized against Test-3arrays (Affymetrix, CA). As these met the criteria (bioC, bioD and cre were present, the 3'/5' ratio of the polyA controls was < 3) the cRNAs were hybridized against Affymetrix HU133A-chips. RNA preparation and hybridization was performed according to the Affymetrix user manual. Raw data were normalized globally and processed with Microarray Suite (MAS 5.0, Affymetrix) and Data Mining Tool (DMT, Affymetrix). The mean of the replicates was calculated and differentially expressed genes between primary trophoblasts and ACH-3P as well as between primary trophoblasts and AC1-1 were selected by the following criteria: fold change ≥ 2 or ≤ -2, change p-value ≥ 0.992 or ≤ 0.008 and at least one signal intensity (control or treatment) >100. Annotations were obtained from NetAffx.

### Functional classification

The 'Gene Ontology Mining Tool' was used to extract the putative biological function of the GeneChip probe sets contained within subclusters as defined by the 'Gene Ontology Consortium' . The Gene Ontology Mining Tool determines the probability that a particular category of 'biological process' is enriched in the individual subclusters calculated using the Chi squared test. Biological Significance was based on the chi-square value, which indicates significant enrichment of a particular biological process within a set of probe sets genes. Differentially expressed genes selected as above were clustered hierarchically using the EpClust software.

### Immuno-separation of HLA-G positive and negative cells

ACH-3P cells were enzymatically detached by accutase (PAA Laboratories, Pasching, Austria) treatment and filtered through a cell sieve in order to obtain a single cell suspension. First trimester primary trophoblast cells were separated immediately after the normal isolation protocol. Cells were separated by incubation with immuno-magnetic beads (Dynabeads M-450) conjugated with anti HLA-G antibody (MEM-G/9; Abcam). The unbound, HLA-G negative, cells and the cells bound to the beads were subsequently cultured in the appropriate medium. The quality of separations was controlled by measuring expression levels of HLA-G (mRNA).

### Semi quantitative RT-PCR

Total RNA was isolated from the HLA-G positive and negative ACH-3P with Trizole. Primers (Table 2) were designed using the public web-page Primer3  and purchased from Ingenetix (Vienna, Austria). The primer-pairs included splicing sites within the amplicon. The mRNA-amount of the ribosomal protein L30 (RPL30) was used as an internal control. 200 ng total RNA was used for the one step RT-PCR kit from Quiagen (Hilden, Germany) according to the manufacturer's instructions. The optimum cycle number was determined for each primer-pair (given in Table 2). The annealing temperature for all primer pairs was 59°C. PCR-products were electrophoresed on 3% agarose gels, documented with the Eagle-Eye™ system (Stratagene, CA) and quantified with the AlphaDigiDoc 1000 (Alpha Innotech, CA) software.

### TNF-α treatment of HLA-G positive and negative ACH-3P cells

One × 10^5 ^HLA-G positive and negative ACH-3P cells (passage 2) were seeded into 12 well plates in DMEM medium with 10% (v/v) FCS. After 24 h, cells were serum starved for 4 h in DMEM without FCS followed by addition of 10 ng/ml human recombinant TNF-α (Sigma). No TNF-α was added to the controls. Cells were cultured in the presence of 20% oxygen for 48 hours at which time RNA was isolated (Trizole) and expression of *RPL30 *and *MMP15 *was measured by RT-PCR. The experiment was performed in quadruplicates.

### TNF-α treatment on first trimester explants

First trimester placenta tissue samples (week 7–8) were rinsed in PBS and dissected into small pieces of approximately 10 mg moist mass. Placental villi were clamped on small polystyrene floats and cultured in 12 well dishes floating in DMEM/F12 (1:1, v/v) (F12, Sigma, Steinheim, Germany) without FCS for 48 h. Explant cultures were incubated in a hypoxy-bench (invivo_2 _1000, Ruskinn Technology, Pencoed, Bridgend, UK) at 37°C under 3% O_2 _and 5% CO_2_. For TNF-α treatment the culture medium was supplemented with 10 ng/ml TNF-α (Sigma). The experiment was run in triplicates.

### Statistical analysis

The results are presented as mean ± standard deviation. After testing for normal distribution Student's t-test was used to test for significant differences. P-values of less than 0.05 were accepted as statistically significant.

## Authors' contributions

UH studied the differences of HLA-G positive and negative cells and drafted the manuscript. CW participated in the design of the study and helped to draft the manuscript. NP carried out the cell preparations, cell culture and zymography. MG did the explant culture experiments and the treatments with TNF-α. UW conducted the invasion assays. HGF and US carried out the cell fusion. CF-H and MH did the karyotyping. AP and AR carried out the FACS analysis. MK, PH and MB did the microarray analysis and participated in the manuscript. PK helped to draft the manuscript and GD participated in the study design and drafted the mansuscript. All authors read and approved the final manuscript.

## Supplementary Material

Additional File 1 **Supplementary Table 1: Downregulated genes** Click here for file

Additional File 2 **Supplementary Table 2: Upregulated genes** Click here for file

Additional File 3 **Supplementary Figure 1: Number of genes – downregulated in JEG-3**. 619 genes higher expressed in primary trophoblasts and ACH3P as compared to JEG-3 were clustered into 6 characteristic functional categories (P < 0.05). The full list of genes is given in the suppl Table 1. **Supplemental Figure 2: Number of genes-upregulated in JEG3**. 213 genes higher expressed in JEG-3 as compared to primary trophoblasts and ACH3P were clustered into 6 characteristic functional categories (P < 0.05). The full list of genes is given in the supplementary Table 2. Click here for file
