# Supplementary material for: The first trimester human trophoblast cell line ACH-3P: A novel tool to study autocrine/paracrine regulatory loops of human trophoblast subpopulations – TNF-α stimulates MMP15 expression
Source: BMC Dev Biol. 2007 Dec 19;7:137. doi: 10.1186/1471-213X-7-137 (PMC2263055; doi:10.1186/1471-213X-7-137)
Supplement: Additional File 3 — Supplementary Figure 1: Number of genes – downregulated in JEG-3. 619 genes higher expressed in primary trophoblasts and ACH3P as compared to JEG-3 were clustered into 6 characteristic functional categories (P < 0.05). The full list of genes is given in the suppl Table 1. Supplemental Figure 2: Number of genes-upregulated in JEG3. 213 genes higher expressed in JEG-3 as compared to primary trophoblasts and ACH3P were clustered into 6 characteristic functional categories (P < 0.05). The full list of genes is given in the supplementary Table 2. [file 1471-213X-7-137-S3.pdf]

Supplementary figures

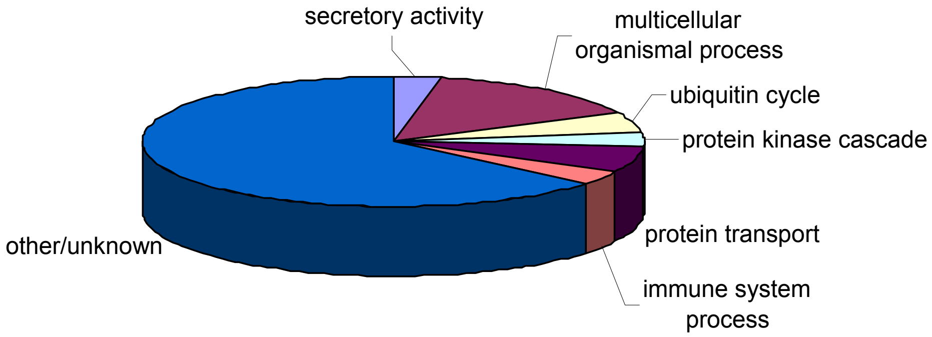

Supplemental Figure 1: Number of genes – downregulated in JEG-3

619 genes higher expressed in primary trophoblasts and ACH3P as compared to JEG-3 were clustered into 6 characteristic functional categories ($P < 0.05$). The full list of genes is given in the suppl Table 1.

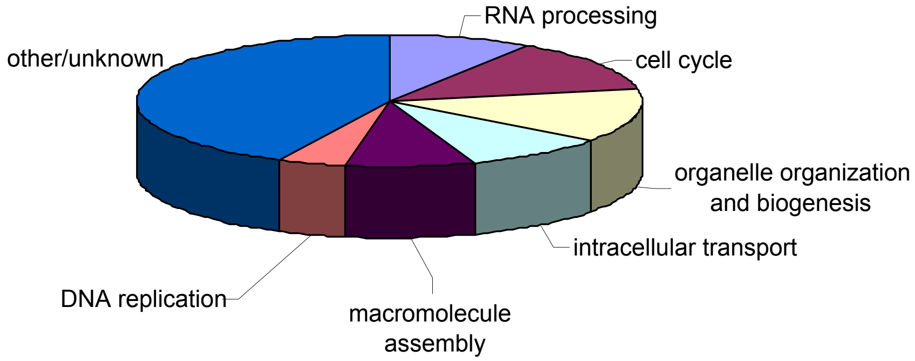

Supplemental Figure 2: Number of genes-upregulated in JEG3

213 genes higher expressed in JEG-3 as compared to primary trophoblasts and ACH3P were clustered into 6 characteristic functional categories ($P < 0.05$). The full list of genes is given in the suppl Table 2.
